# Bladder Uptake of Liposomes after Intravesical Administration Occurs by Endocytosis

**DOI:** 10.1371/journal.pone.0122766

**Published:** 2015-03-26

**Authors:** Bharathi Raja Rajaganapathy, Michael B. Chancellor, Jayabalan Nirmal, Loan Dang, Pradeep Tyagi

**Affiliations:** 1 Department of Urology, William Beaumont Hospital Research Institute, Oakland University William Beaumont School of Medicine, Royal Oak, Michigan, United States of America; 2 Department of Urology, University of Pittsburgh, Pittsburgh, Pennsylvania, United States of America; 3 Eye Research Institute, Oakland University, Rochester, Michigan, United States of America; Okayama University, JAPAN

## Abstract

Liposomes have been used therapeutically and as a local drug delivery system in the bladder. However, the exact mechanism for the uptake of liposomes by bladder cells is unclear. In the present study, we investigated the role of endocytosis in the uptake of liposomes by cultured human UROtsa cells of urothelium and rat bladder. UROtsa cells were incubated in serum-free media with liposomes containing colloidal gold particles for 2 h either at 37°C or at 4°C. Transmission Electron Microscopy (TEM) images of cells incubated at 37°C found endocytic vesicles containing gold inside the cells. In contrast, only extracellular binding was noticed in cells incubated with liposomes at 4°C. Absence of liposome internalization at 4°C indicates the need of energy dependent endocytosis as the primary mechanism of entry of liposomes into the urothelium. Flow cytometry analysis revealed that the uptake of liposomes at 37°C occurs via clathrin mediated endocytosis. Based on these observations, we propose that clathrin mediated endocytosis is the main route of entry for liposomes into the urothelial layer of the bladder and the findings here support the usefulness of liposomes in intravesical drug delivery.

## Introduction

Intravesical therapy is the routine first-line treatment for effectively delaying or preventing the recurrence of bladder cancer [1]. This route of drug administration has also shown tremendous promise in the treatment of interstitial cystitis/painful bladder syndrome (IC/PBS) [2] and overactive bladder [3] to justify investments for further improvements [4]. Intravescial route of drug administration is preferred in conditions which are refractory to conventional treatment by oral route. Orally administered agents are often unable to create effective luminal drug concentration due to low urinary excretion of drugs, which justifies bladder instillation [5]. Replacement of glycosaminoglycan (GAG) layer by intravesical administration of hyaluronic acid has been successfully tried in IC/PBS [6].

The urinary bladder has evolved to form an effective epithelial barrier to hold urine which carries toxic substances. The barrier function exerted by the epithelium of urinary bladder (urothelium) has typical umbrella cells attached by tight junction proteins and concealed by impenetrable plaques and anti-adherent mucin layer. Low permeability of the urothelium limits the penetration of the instilled drug across the urothelium [7] which allows administration of agents that may be too toxic for systemic administration. Direct instillation or cystoscope guided injection of potentially toxic drugs such as botulinumtoxin or resiniferatoxin in the bladder can achieve maximal therapeutic benefit at the target site without systemic toxicity [8]. Several drugs including lidocaine, heparin, dimethyl sulfoxide, chondroitin sulfate, hyaluronic acid have been shown to demonstrate benefit for lower urinary tract symptoms (LUTS) via intravesical route, but a similar benefit in LUTS is not derived from their systemic administration.

Intravesical therapy also holds the potential to facilitate the separation of therapeutic effect from side-effects by involving a diverse array of novel chemical, pharmacological, and formulation strategies. However, drug delivery by intravesical route is constrained by the impermeability of the urothelium and the need for frequent administration. Instilled drug solutions become diluted with urine and get washed out of the bladder during voiding, necessitating repeated infusions of the drug. Therefore, there is ongoing search for drug delivery methods to improve intravesical drug delivery [9].

Of all the drug delivery platforms, liposomes have been approved by US Food and Drug Administration for the safe and effective delivery of several drugs [10]. In recent years, empty liposomes have been instilled as a potential therapy for interstitial cystitis [11,12]. Liposomes have vesicular structures consisting of an aqueous core surrounded by a lipid bilayer [13], which allow delivery of both lipophilic drugs such as capsaicin and hydrophilic drugs such as botulinum toxin. The therapeutic advantage derived from use of liposomes as a drug carrier has been previously demonstrated in human and animal studies [1,9,11,12]. However, the exact cellular mechanism underlying liposome uptake by bladder remained unclear. Endocytosis pathway was shown to be a possible major contributor to the cellular uptake of liposomes [1]. Hence we investigated the possible role of endocytosis in the uptake of liposomes by urothelium.

## Materials and Methods

### Materials

Dulbecco’s Modified Eagle Medium (DMEM) was obtained from GIBCO, BRL (Carlsbad, CA, USA). N-[12-[(7-nitro-2-1,3-benzoxadiazol-4-yl)amino]dodecanoyl] (NBD) was purchased from Avanti Polar Lipids (Birmingham, AL, USA). The chemicals Annexin V Apoptosis detection (AAD), Methyl-β-cyclodextrin (mhCD), Chlorpromazine (CPZ) and Dithionite were obtained from Sigma Aldrich, St. Louis. Phosphate-buffered saline (PBS) was purchased from Fisher Scientific. Normal human urothelium UROtsa cells were kindly provided by Dr. Scott Garrett (University of North Dakota, USA) and cultured in DMEM (Gibco) without phenol red [14].

### Preparation of liposomes

50 mg of lipids obtained from Avanti Polar was dissolved in a chloroform methanol mixture (3:1) which was dried into a thin film inside walls of a round bottom flask using steady stream of nitrogen. The round bottom flask with lipid was kept overnight under vacuum to remove any traces of organic solvent. Then 5 mL of colloidal gold nanoparticle (10 nm) was added to hydrate the dried lipid followed by vortex and sonication at 65°C. The round bottom flask was kept at -80°C for 1 h and thawed using ice for 30 min and again vortexed and sonicated at 65°C. The freeze thaw cycle was repeated 3 times to procure the liposome encapsulated gold, which was centrifuged at 2000rpm for 5 min and pellet resuspended in sterile water for use in both *in vitro* and *in vivo* studies for TEM. The final lipid concentration of liposomes in the experiments was similar that used in studies employing liposomes as a carrier for botulinum toxin [15].

### In vitro urothelium cell uptake study

UROtsa cells (1x10^5^) were plated in 100 mm petridishes and grown for 2 days in Dulbecco’s modified Eagle’s medium (DMEM) containing 5% v/v fetal bovine serum at 37°C in 5% CO_2_ atmosphere. Cells were incubated with or without liposomes in serum free DMEM media for 2 h either at 37°C or at 4°C. After 2 h incubation, cells were washed with plain media and subjected for TEM study.

### In vivo bladder uptake study

This study was carried out in strict accordance with the recommendations in the Guide for the Care and Use of Laboratory Animals of the National Institutes of Health. The protocol was approved by the Beaumont Research Institute (Royal Oak, MI, USA) Animal Care Committee (IACUC approval number: AL-12-07). Female Sprague Dawley rats were procured from Charles River Laboratories, MA. Rats were divided into 2 groups; group 1 instilled with empty liposomes (n = 4) and group 2 treated with liposomes encapsulated gold (lipo-gold) (n = 4). Under isoflurane anesthesia, rat bladder was emptied of urine, and then a catheter was inserted through the urethra and instilled with 0.5 mL of liposome or lipo-gold into the bladder for 30 min. After instillation, animals were allowed to recover and returned to their cages. Animals were sacrificed 8 h after instillation by administering sodium pentobarbitone 120 mg/kg. Rat bladders were washed with normal saline, filled with 0.5 mL of fixative solution (1% osmium tetroxide in 0.1 M cacodylate), harvested and then placed in fixative solution for TEM study.

### TEM Study

UROtsa cells and bladder specimens were rinsed with 0.2M Cacodylate pH 7.4 for 5 min and fixed with 2% glutaraldehyde in 0.1M Cacodylate. Specimens were post fixed using 1% osmium tetroxide in 0.1M cacodylate for 1 h at 4°C. Fixed specimens were given a wash with 7% sucrose overnight and dehydrated for 15 min at graded concentrations (50, 70, 90 & 100%) of ethanol and then incubated in propylene oxide for 10 min followed by infiltration of specimen with 1:1 (propylene oxide:812 epoxy resin) for 1 h. Subsequent steps involved embedding in block and polymerization at 60°C overnight. Ultra-thin sections were cut with Ultra-microtome RMC MT-7000, Tucson, AZ, which was negatively stained with uranyl acetate with silver enhancer. Images were acquired using Morgagni 268 Transmission Electron Microscope FEI Company, Hillsboro, OR.

### Flow cytometry

UROtsa cells were seeded in 6-well plates (1x10^5^) for approximately 2–3 days to achieve 70% confluency. Cells were incubated in phenol red free DMEM with liposomes containing fluorescent label NBD for 60 min. After incubation, cells were washed once with 2 mL PBS, collected by trypsinisation, pelleted, washed once in 1 mL PBS and resuspended in 0.3 mL PBS. The cell suspension was analyzed within 30 min. In the case of NBD-labeled liposomes, an intermediate step prior to trypsinization was done. This involved addition of 50 μL of a 1.5M dithionite solution to the cells for 5 min and the cells were washed 3X with PBS. For blocking experiments, cells were incubated with the inhibitors Chlorpromazine (CPZ) (inhibitor of clathrin mediated endocytosis) and Methyl-β-cyclodextrin (mhCD) (inhibitor of caveolae mediated endocytosis) prior to incubation with liposomes.

Flow cytometric analysis for fluorescent signal from liposomes was performed using a 4-color FACS-Calibur (Becton Dickinson, Heidelberg, Germany) equipped with an argon laser exciting at a wavelength of 488 nm. For each sample, 10000 events were collected by list-mode data that consisted of side scatter, forward scatter and fluorescence emission centered at 530 nm (FL1) and 585 nm (FL2), respectively. For 7-Annexin V Apoptosis detection (AAD), a long-pass filter with a cutoff of 670 nm (FL3) was applied. The fluorescence was collected on a logarithmic scale with a 1024 channel resolution. Cell Quest Pro software (Becton Dickinson, Heidelberg, Germany) was applied for the analyses.

### Statistical analysis

Statistical analysis was performed using GraphPad Prism, 4.03 (San Diego). One way analysis of variance (ANOVA) followed by Dunnett’s post hoc used for other parameters. Data was expressed in means ± S.E.M. P < 0.05 was fixed as the statistical significance criterion.

## Results

### In vitro study

**Fig 1:**
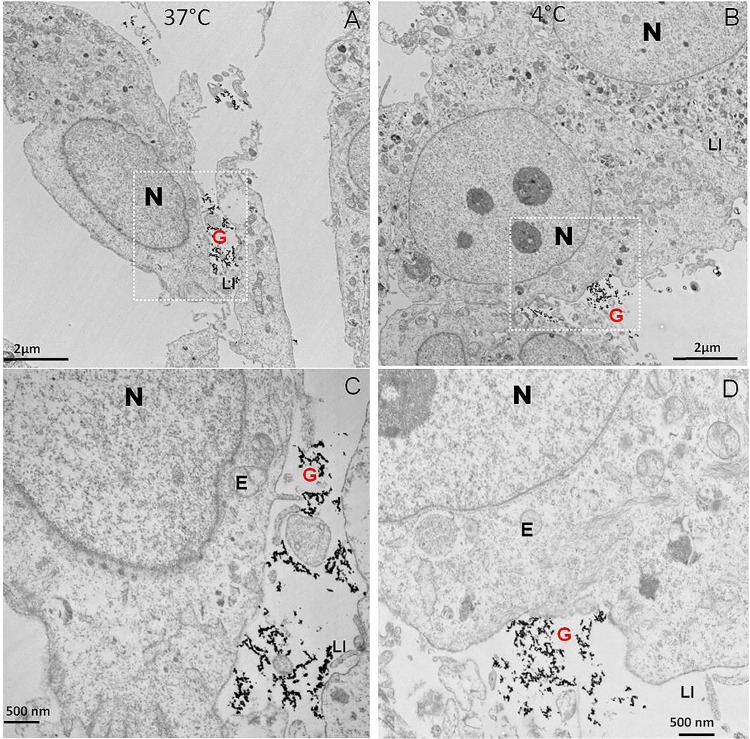
TEM micrographs of UROtsa cells demonstrate the lack of any internalization of colloidal gold particles in absence of liposomes by absence of any dark grains inside the cells. Compared to faint to dark grey color of uranyl acetate staining acquired by other cell organelles and electron dense gold particles appear as dark grains. UROtsa cells incubated at either 37°C (Panel A&C) or at 4°C (Panel B&D) showed similar absence of any dark grains inside the endosome marked by E in all the panels to indicate uptake was independent of temperature. Inscribed area of panel A and B in white rectangle is magnified 3 times further in panel C and D, respectively. UROtsa cells have prominent large nucleus shown by N and cells are marked by finger like projections called lateral interdigitation (shown by LI) for connecting with adjacent cells. Plain gold is marked by red colored G, Magnification is shown by the scale bar in each panel.

Ultrastructural studies of UROtsa cells showed that cells lie adjacent to one another in a monolayer arrangement and are connected to one another with finger like projections called as lateral interdigitation (LI). The size of nucleus in UROtsa cell is comparatively larger with higher nuclear to cytoplasmic ratio. UROtsa cells incubated with colloidal gold particles in absence of liposomes did not internalize any of the gold particles as indicated by dark grains lying outside the cell on EM image representing gold particles (G) (Fig. 1). Compared to faint to dark grey color of uranyl acetate staining acquired by other cell organelles, the electron dense gold particles appear as dark grains. Inscribed area in panel A and B is magnified 3 times further in panel C and D, respectively to show a single cell. The cellular uptake of gold particles was not affected by temperature as evident from the EM images of cells taken after incubation at 37°C (Panel A&C) or at 4°C (Panel B&D) for 2 h.

**Fig 2:**
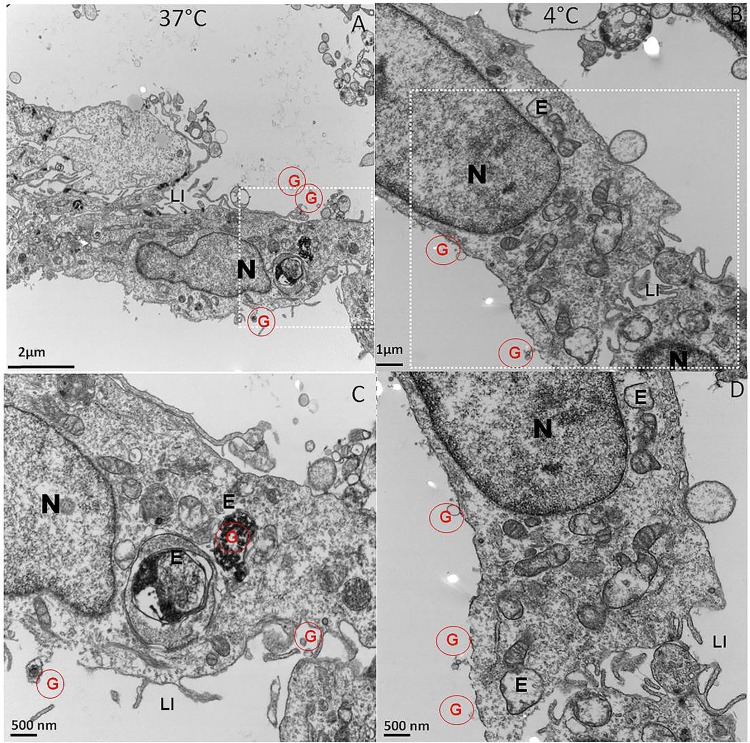
TEM micrographs showing endocytosis mediated uptake of liposome encapsulated gold marker at 37°C (Panel A&C). Higher magnification image of inscribed area from panel A showed that vesicle like structures in endosomal compartment (marked by E) contained cluster of electron dense dark particles inside a single cell (Panel C). In contrast, only extracellular binding of liposomes containing dark grains was observed at 4°C (Panel B) and corresponding higher magnification image (Panel D) showed that vesicle like structures in a single cell were devoid of dark gold particles, which indicates absence of internalization due to temperature dependent inhibition of endocytosis. Gold encapsulated in liposomes is marked by red colored G inside a red circle, nucleus is marked by N, endocytic vesicles are marked by E and finger like projections called as lateral interdigitation are marked by LI. Inscribed area of panel A and B in white rectangle is magnified further in panel C and D, respectively and magnification is shown by scale bar in each panel.

In contrast, EM images of UROtsa cells incubated with liposomes encapsulating gold particles at 37°C showed cluster of dark gold particles inside the cell and not outside the cell membrane (Fig. 2A). Corresponding higher magnification of the image showed that dark grains of gold were associated with vesicle like structures in an endosomal compartment (E) of UROtsa cells (Fig. 2C). Incubation of UROtsa cells with liposomes encapsulating gold particles at 4°C showed absence of dark grains inside the cell and only extracellular binding of liposomes containing dark grains of gold was seen (Fig. 2B). Corresponding higher magnification in Panel D showed that vesicle like structures were devoid of dark gold particles indicating absence of internalization and the temperature dependence for the cellular uptake of encapsulated gold particles. Since temperature had no effect on the internalization of gold particles (Fig. 1), it can be inferred that liposomes as a carrier is necessary for the endocytosis to occur.

### In vivo study

**Fig 3:**
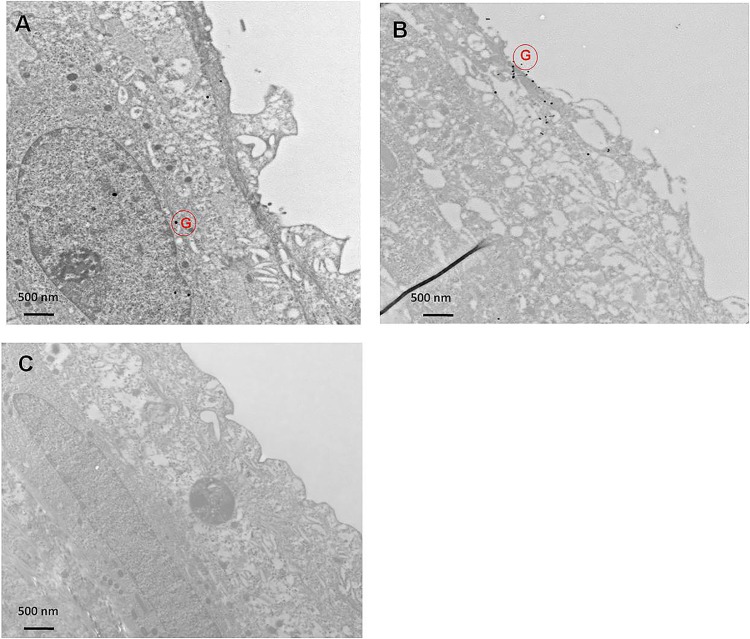
TEM micrographs showing endocytosis mediated uptake of liposome encapsulated gold in rat bladder (A) as revealed by electron dense dark grains consistent with uptake of gold across the urothelium. (B) Dense black grains binding to the cell surface were noted in rat group instilled with colloidal gold in absence of liposomes. Gold encapsulated in liposomes are marked by red colored G inside a red circle and plain gold is marked by yellow G in the images. (C) Untreated rat bladder is marked by (absence of dark black gold grains. Magnification of 8900x was used in all the images and is shown by the scale bar.

Rat bladder was instilled with encapsulated liposome showed the uptake of gold across the urothelium (Fig. 3A); whereas the bladder instilled with plain gold showed the gold particles lying outside the tissue region of sections (Fig. 3B). The untreated bladder is shown as control image (Fig. 3C). The results support the evidence regarding the endocytosis of liposomes in bladder [1].

### Flow Cytometry

**Fig 4:**
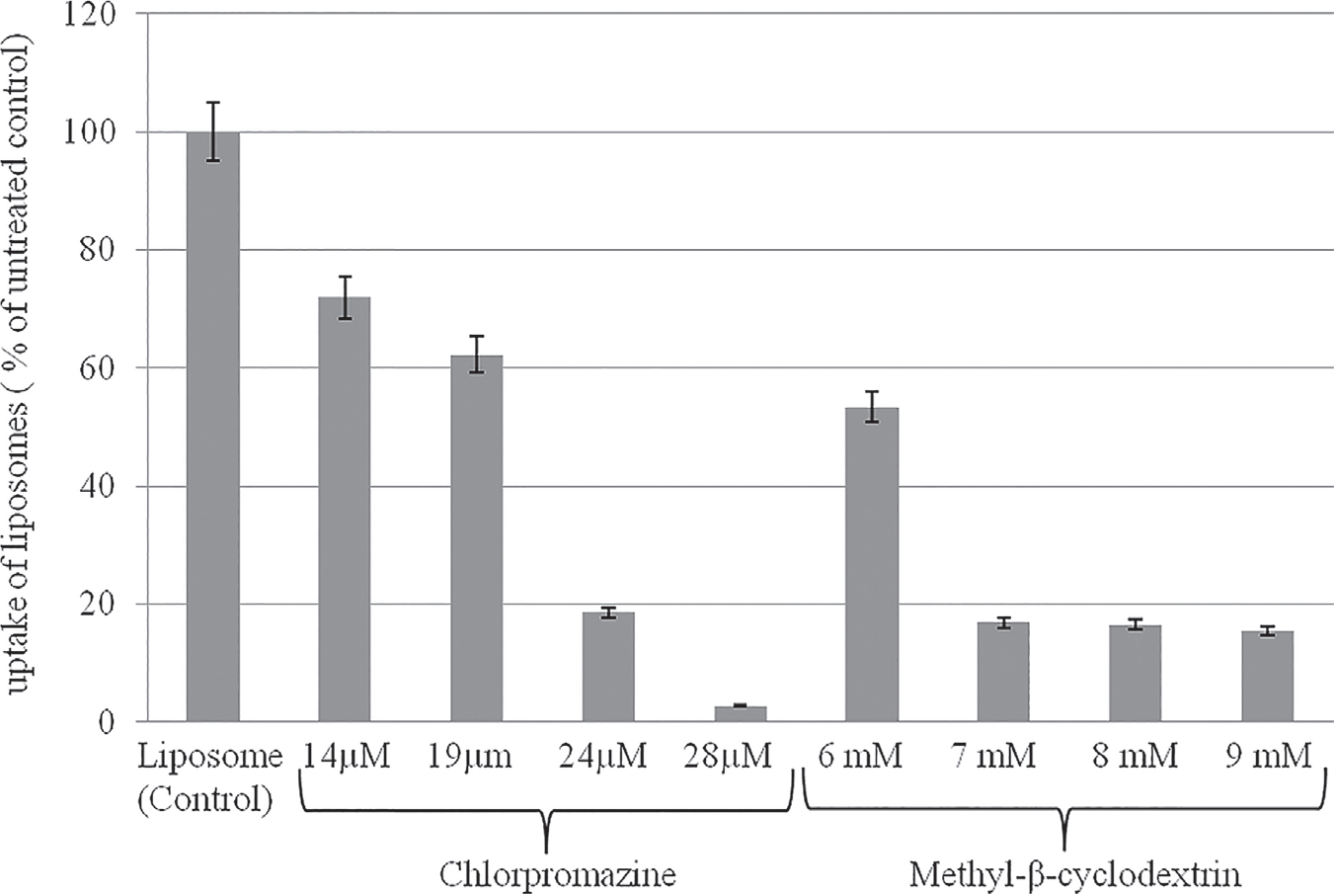
Effect of different endocytic inhibitors on cellular uptake of fluorescent liposomes. Flow cytometry analysis suggest clathrin mediated endocytosis as the dominant pathway for the internalization of fluorescently labeled liposomes by UROtsa cells at 37°C. Chlorpromazine (inhibitor of clathrin) dose dependently inhibited the internalization as cell fluorescence from externally bound non-internalized liposomes was quenched by sodium dithionite. Fluorescence intensity of liposomes in cells untreated with inhibitors was taken as 100%.

The different endocytosis mechanisms operational in liposome uptake were studied with the help of different inhibitors. In this study, we used a series of pharmacological inhibitors: CPZ concentration range from 14 to 28 μM and mhCD concentration range from 6 to 9 mM to inhibit clathrin and caveole mediated endocytosis, respectively. Pre incubation of UROtsa cells with CPZ for 60 min caused partial blockade of fluorescent liposome uptake at 24 μM and complete blockade at 28 μM. As shown by flow cytometric analysis, the uptake of liposomes in UROtsa cells was also reduced by 50% after a preincubation period of 60 min with mhCD (Fig. 4), but complete blockade was not achieved as increased cell death limited the options of using a higher concentrations of mhCD or longer preincubation times.

## Discussion

In the present study, the endocytosis mechanism of liposome uptake was carefully studied by functional and ultrastructural studies performed on urothelium cell line. Earlier studies using liposomes constituted with uptake tracking molecules indicated possible involvement of endocytosis in the cellular uptake of the liposomes [16]. Liposomes have been widely used as drug carriers for systemic administration of chemotherapeutic agents [17,18] to achieve the outcome of improved pharmacokinetics and reduced toxicity [18–20]. The success of liposomes in the clinic has been attributed to the nontoxic nature of the lipids used in their formulation. Previous studies from our group have reported that incorporation of tacrolimus into liposomes can be developed as an effective treatment for hemorrhagic cystitis [11]. Liposomes improved the pharmacokinetics of liposomal tacrolimus by maintaining higher drug concentration inside the bladder tissue compared to the plain tacrolimus [21]. However, the mechanism of bladder uptake for instilled liposomes was unclear.

**Fig 5:**
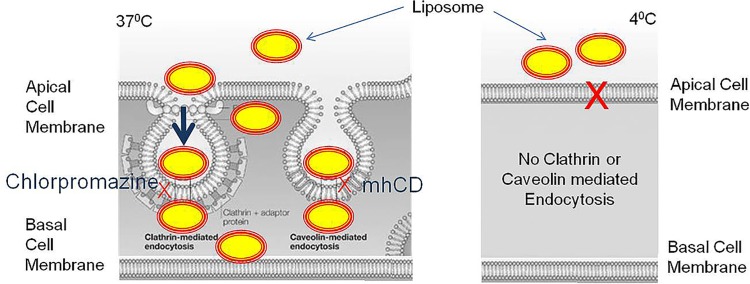
Illustration depict temperature dependent endocytoic uptake of liposome encapsulated gold (Lipo-gold). The cellular process of clathrin and caveolin mediated endocytosis are energy dependent and therefore only occur at 37°C and are inhibited at 4°C. Compared to mhCD, chlorpomazine was more efficient in blocking endocytosis of liposomes, which indicates a predominance of clathrin mediated endocytosis as a mechanism of endocytosis.

Absence of liposome internalization at 4°C in TEM images indicates the need of energy dependent endocytosis as the primary mechanism of entry for liposomes into urothelium. UROtsa cells incubated with liposome at 37°C showed endocytic vesicles containing liposomes as indicated by electron dense gold particles appearing as dark grains. Images suggest that upon binding of liposomes with cell membrane, the endocytic internalization of lipo-gold would have occurred and then released from the endosomal vesicles [22] (Fig. 5). The liposomes wrapped by the endosomal membrane get destabilized due to intermingling of lamellar phase-perturbing lipids with the endosomal membrane [23]. Findings obtained at 4°C are consistent with decreased ability of liposomes composed of non-ionic lipids to fuse with endocytic vesicles at this temperature [22]. Findings from cellular experiments were reproduced in rat bladder, where TEM images of rat bladder 8 h after instillation of lipo-gold showed several gold grains inside the urothelium. Intracellular gold was missing in rat instilled with plain gold. Findings suggest that bladder uptake of liposomes involved endocytosis. From this, it is evident that the transport of drugs (represented by colloidal gold) instilled in the bladder is dependent upon the pathway of entry preferred by carrier over mechanism preferred by the encapsulated drugs in the liposomes [24] (Fig. 5).

Several aspects of internalization regarding different forms of endocytosis involved in liposome uptake are illustrated in Fig. 5. It is accepted that all forms of endocytosis, whether dependent or independent of caveolin or clathrin mediated pathways do require energy, which therefore explains the inhibition of liposome uptake by cells incubated at 4°C. Complete blockade of fluorescent liposome uptake by CPZ suggests that UROtsa cells internalize liposomes via clathrin mediated endocytosis. CPZ interacts with clathrin of the coated pits and causes their loss from the surface membrane, which therefore makes CPZ an important tool for studying clathrin mediated endocytosis [25]. Preincubation with CPZ caused a dose dependent inhibition of fluorescent liposome uptake by UROtsa cells, which suggests the preponderance of clathrin mediated endocytosis in internalization of liposomes by UROtsa cells. Predominance of clathrin mediated endocytosis of liposomes is also consistent with the accumulation of dark gold grains in the subcellular organelle of endosome in the TEM images.

Interestingly, a role for caveolar endocytosis was previously reported in the liposome uptake of cultured HeLa cells [16] and therefore, we also presumed a role for caveolar endocytosis in liposome uptake by UROtsa cells as illustrated in Fig. 5. We used water soluble mhCD for inhibiting caveolae mediated endocytosis, as it has been used in other published studies [26,27]. In our experiments, mhCD only partially blocked the fluorescent liposome uptake by UROtsa cells, which suggest that caveolin mediated endocytosis was less dominant than clathrin mediated endocytosis for liposomes. Moreover, it is known that mhCD forms soluble inclusion complexes with cholesterol and depletes cholesterol from the plasma membrane, which can compromise cholesterol rich microdomains involved in both caveolae as well as clathrin mediated endocytosis. This effect of mhCD on membrane structure may in part explain the absence of a dose dependent inhibition of fluorescence uptake seen in cell preincubated with mhCD. The FACS analysis and accumulation of liposomes encapsulated gold in endosome (TEM images) together support the dominance of clathrin mediated endocytosis over caveolar endocytosis for liposomes in UROtsa cells.

As shown in Fig. 5, UROtsa cells may employ several diverse energy dependent processes in parallel for liposome uptake. Based on published reports, one or other process may dominate depending on attributes of liposomes and cells tested. In conclusion, the findings of our study demonstrate *in vitro* and *in vivo* evidence for endocytosis as the mechanism of liposome uptake in bladder.
